# Quantitative Cryo-Electron Tomography

**DOI:** 10.3389/fmolb.2022.934465

**Published:** 2022-07-06

**Authors:** Paula P. Navarro

**Affiliations:** ^1^ Department of Molecular Biology, Massachusetts General Hospital, Boston, MA, United States; ^2^ Department of Genetics, Harvard Medical School, Boston, MA, United States

**Keywords:** cryo-electron tomography, *in situ* structural biology, quantitative cryo-electron tomography, image processing, cell biology

## Abstract

The three-dimensional organization of biomolecules important for the functioning of all living systems can be determined by cryo-electron tomography imaging under native biological contexts. Cryo-electron tomography is continually expanding and evolving, and the development of new methods that use the latest technology for sample thinning is enabling the visualization of ever larger and more complex biological systems, allowing imaging across scales. Quantitative cryo-electron tomography possesses the capability of visualizing the impact of molecular and environmental perturbations in subcellular structure and function to understand fundamental biological processes. This review provides an overview of current hardware and software developments that allow quantitative cryo-electron tomography studies and their limitations and how overcoming them may allow us to unleash the full power of cryo-electron tomography.

## 1 Introduction

Determination of the structure of biomolecules is torn between resolution and physiological context, aiming to obtain the three-dimensional (3D) architecture of macromolecules. Structural biology methods such as X-ray crystallography (X-ray), nuclear magnetic resonance spectroscopy, and more recently, single particle analysis (SPA) cryo-electron microscopy (cryo-EM) have been at the forefront of structural biology with important implications in the fields of pharmacology and biomedical research by determining the atomic structure of isolated macromolecules (Garman, 2014; Kühlbrandt, 2014).

A major goal of structural biology is to visualize macromolecular complexes in action inside the cell at high resolution. However, most solved structures come from methods based on purified and concentrated proteins extracted from cells that overexpress them. A compelling question here is how the structure of such isolated macromolecules differs from that occurring inside cells under normal expression levels. Biological processes are rarely attributed to the individual action of one macromolecule but to their orchestrated action with binding partners and subcellular elements, pointing out the need of methods allowing structural studies of unperturbed cellular environments. Pioneering cryo-electron tomography (cryo-ET) work from the Baumeister (Medalia et al., 2002; Lucić et al., 2005), Jensen (Tocheva et al., 2010; Gan and Jensen, 2012) and Briggs (Briggs, 2013; Wan and Briggs, 2016) labs among others have positioned cryo-ET as a promising structural biology method. This has opened a new field of research: *in situ* structural biology (Asano et al., 2016).

**FIGURE 1:**
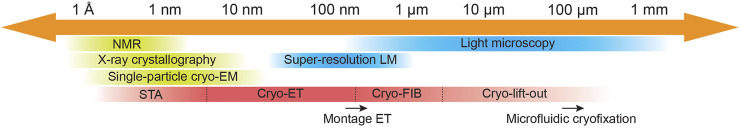
Cryo-electron tomography imaging across scales. Schematic representing resolution limitations for commonly used imaging methods in cellular and structural biology indicating resolution gaps and potential bridges. Cryo-electron tomography was initially restricted to image viruses, small bacteria cells, and thin regions of mammalian cells (e.g., axons and lamellipodia) at nanometer resolution. Software developments for pre-processing of tilt-series as beam-induced motion correction, tilt-series alignment refinement and 3D CTF-correction, and for STA as alignment refinement and classification, have allowed *in situ* structure determination in cryo-ET data by STA. On the other hand, technologies such as cryo-FIB milling and cryo-lift-out have allowed imaging of cells and multicellular organisms *in situ*, respectively. Relevant ongoing developments that help expand cryo-ET are pointed out by a black arrow at their corresponding length scale area. Montage ET allows image acquisition of larger field of views (FOV) without compromising resolution at the cost of elongated acquisition times (Peck et al., 2021; Yang et al., 2022). While microfluidic cryofixation of larger specimens during live-cell microscopy imaging is a promising tool to explore the opportunities for correlative light and electron microscopy studies (Fuest et al., 2019).

Cryo-ET reflects the duality between resolution and physiological relevance inherent to the field of structural biology. Cryo-ET is a structural biology method capable of visualizing the 3D structure of macromolecules in their native context. Current software and hardware developments are focused on stretching both ends of the scale length that cryo-ET initially was restricted to, aiming to cover more research areas and applications by conquering current resolution gaps among imaging methods (Figure 1). Subtomogram averaging (STA), a 3D image processing technique, has pushed one end of the length scale by enabling atomic structure determination by cryo-ET (Schur et al., 2016). At the other end of the scale, technologies such as cryo-focus ion beam (cryo-FIB) milling (de Winter et al., 2013; Rigort and Plitzko, 2015) and cryo-lift-out (Schaffer et al., 2019; Parmenter and Nizamudeen, 2021), allowed the imaging of multicellular organisms and tissues. This positions cryo-ET not only to complement other structural methods, but to buttress results from other imaging modalities (live-cell imaging, light microscopy (LM) imaging) as well as experimental data obtained from molecular biology techniques (e.g., genetic perturbation, CRISPR/Cas9) by visualizing key biological events captured in action at the molecular level. Likewise, this crucial information can be complemented with target functional data to provide a complete description of cellular processes.

In this review, the potential of quantitative cryo-ET for biological applications is first exposed by describing the main advances that are pushing cryo-ET and transforming it into a research field on its own while opening powerful applications entangled with translational biomedical research. In the next sections, the main limitations of the method are summarized: thickness and the trade-off between resolution and field of view (FOV). Finally, a section remarks the need of practitioners to carefully evaluate the convenience of using cryo-ET in their research projects since cryo-ET might not necessarily be the fittest choice.

## 2 Quantitative Cryo-Electron Tomography

Cryo-electron tomography is an imaging method that visualizes macromolecules in preserved cellular contexts at high resolution. In cryo-ET, the specimen is physically rotated inside the microscope during acquisition. Thus, a set of 2D projections from multiple angles is collected, also referred to as tilt-series. The tilt-series is computationally aligned and reconstructed to yield a 3D volume, a tomogram (De Rosier and Klug, 1968). Hundreds to thousands of copies of target macromolecular complexes are contained in tomograms. These copies are extracted into smaller subtomograms, which are called “particles” in STA. Subtomograms are located, extracted, computationally aligned to each other, and averaged to retrieve a signal-enhanced 3D structure of the macromolecular complex of interest at high resolution, even at atomic resolution (Schur et al., 2016; Wan and Briggs, 2016; Leigh et al., 2019).

**FIGURE 2:**
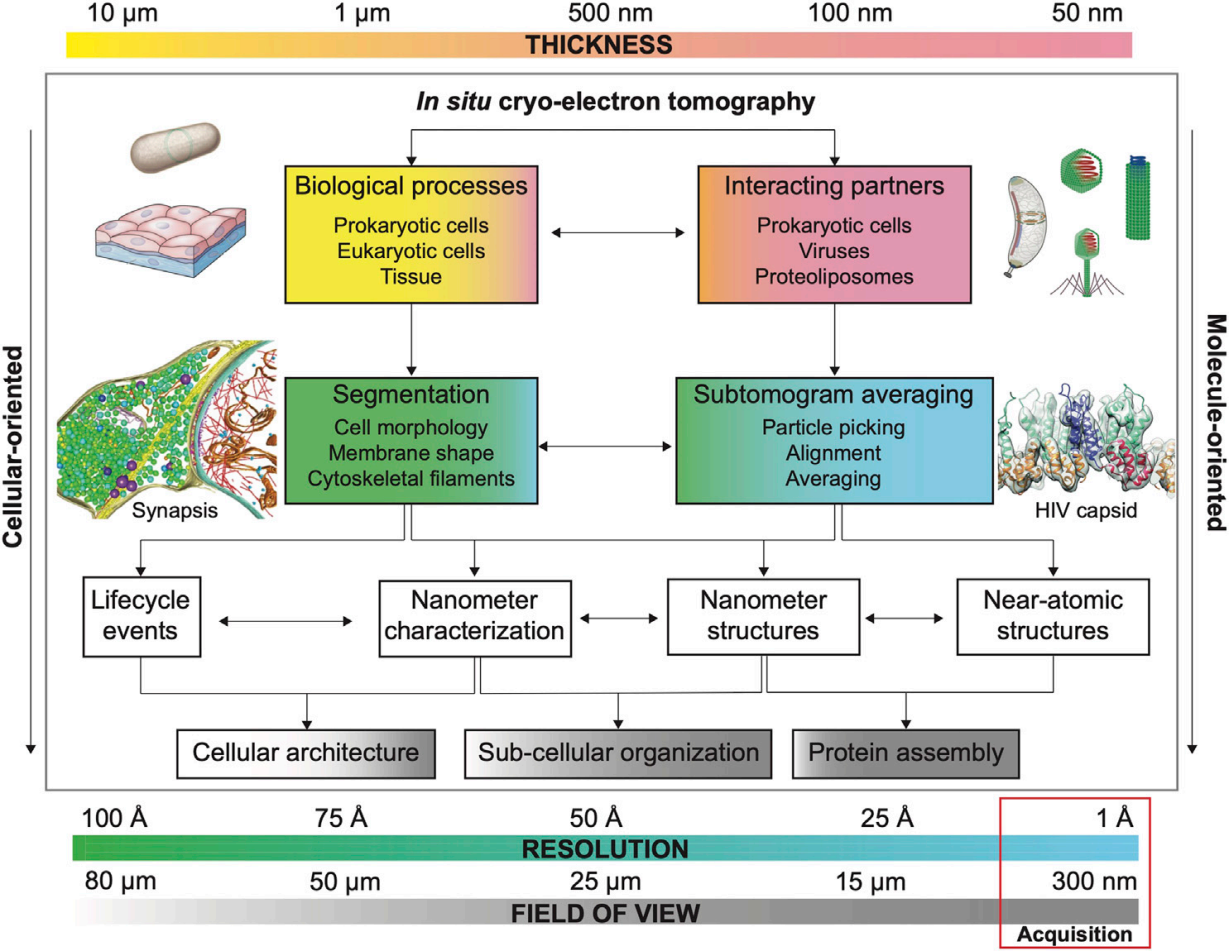
Main considerations for quantitative cryo-ET. Each biological sample possesses an inherent thickness. Sample thickness conditions imaging strategies in cryo-ET (FOV, resolution and need of additional thinning methods, see Section 3). Common FOV and resolution for cryo-ET data acquisition are highlighted by a red box. The combination of these three factors (thickness, FOV and resolution) defines research strategies to answer specific biological questions whose resolution requirements depend on the visualization of size-dependent targets as biological processes (e.g., cell and organellar morphology) and interacting molecular partners (e.g., viral capsid and microtubular structure). Usually, projects aiming to characterize biological processes prioritize FOV over resolution to capture large features while projects that aim to determine the structure of a specific macromolecule by STA *in situ* structure determination prioritize resolution over FOV (number of particles). Segmentation strategies (based on denoised cryo-electron tomograms) are important to understand biological processes while STA is essential for structure determination in projects where interacting partners and molecular structure determination are envisioned. However, segmentation and STA are commonly needed on both types of projects, albeit with different resolution expectations (see Section 2). Example of bacterium adapted from Erickson and Anderson (2010) and tissue sample adapted from Siyavula; example of bacterium (*Caulobacter crecentus*) adapted from ed from Govers and Jacobs-Wagner (2020); example of viruses and phage adapted from from wikipedia by Anderson F. Brito; example of synapsis adapted from Tao et al. (2018); example of HIV capsid adapted from Schur et al. (2016).

Besides protein structure determination *in situ*, the power of cryo-ET lies in its ability to provide contextual information of the cellular environment where macromolecules act. There are two ways of studying the contextual information: molecule-oriented and cellular-oriented. The molecule-oriented approach aims to perform *in situ* structure determination to assess the effects of perturbations in the structure of a macromolecule (Watanabe et al., 2020). The cellular-oriented approach studies the environment to determine the effect of protein dysfunction in the architecture and dynamics of the biological system (Navarro et al., 2021), e.g., organelle and cell morphology. Although with different ultimate goals, both approaches are interrelated and can greatly benefit from the quantitative characterization of the system to fully understand biological processes (Figure 2).

Quantitative cryo-ET can provide a holistic description of the ultrastructural characteristics underlying biological processes and cellular ultrastructures. This structural information can be complemented with functional assays and live-cell imaging to obtain a complete characterization of biological processes. Due to its resolution, cryo-ET is well-positioned to not only buttress experimental data obtained by genetic experiments and LM imaging, but to unravel the cellular landscape. To provide a quantitative characterization, we need sufficiently large datasets (large N numbers) which are hard to obtain by cryo-ET. Large-scale analyses are hindered by a complex pipeline inherent to cryo-ET with bottlenecks in almost each step (e.g., specimen thinning methods, see Section 3), the complexity of cellular data—crowded environments, low signal-to-noise ratio (SNR) of cryo-electron tomograms— and the difficulty of extracting and parameterizing biological features meaningfully. Thus far, cryo-ET is predominantly used as a qualitative method where few tomograms are sufficient for structure determination and to visualize subcellular features, while its power as a quantitative method is not yet completely exploited. Importantly, larger dataset sizes can compensate for the local variation in tomogram quality (due to regions of uneven ice thickness and/or deposition of crystal ice contamination) while smaller dataset sizes might lead to aleatory parameterization errors when analyzing tomogram features, affecting the analysis and results.

### 2.1 Advances Towards Quantitative Cryo-ET

Advents in molecular biology techniques such as CRISPR/Cas9 enable editing target gene sequences in the genome of cells to study a large amount of genetically-modified mutant lines (Charpentier, 2015). Here, cryo-ET can be applied as a quantitative method to identify subcellular and protein structural changes coupled to cell function. Advances in high-throughput tomographic data acquisition are enabling the acquisition of larger datasets faster while computational tools are necessary to automate the processing of cryo-ET data and maximize the extraction of key biological information. Despite the tremendous progress made, quantitative visualization of cell tomograms into organelles and proteins remains a challenging task. Cryo-electron tomograms exhibit inherently low contrast and artifacts introduced by limited angular sampling during imaging (the missing wedge problem). Altogether, cryo-ET involves a difficult and time-consuming pipeline where expert user intervention is mandatory to achieve quality results.

This section focuses on current software developments beneficial for the wide-range analysis of cryo-ET datasets. Then, the three key aspects to enable quantitative analysis of cryo-electron tomograms are exposed: 1) how to combat low SNR—denoising; 2) how to extract, position, and characterize large cellular geometries as organelles, membranes, and filaments by segmentation; and 3) how to maximize the number of extracted particles confidently—template matching.

Faster acquisition schemes (Chreifi et al., 2019; Eisenstein et al., 2019; Schorb et al., 2019; Bouvette et al., 2021) and automation of pre- and post-processing for cryo-ET (motion correction, dose weighting, CTF estimation and correction, tilt-series alignment, tomogram reconstruction, and STA) (Winkler and Taylor, 2013; Mastronarde and Held, 2017; Navarro et al., 2020; Burt et al., 2021; Scaramuzza and Castaño-Díez, 2021; Tegunov et al., 2021) are detailed elsewhere.

#### 2.1.1 Case Examples

Many studies have taken advantage of quantitative cryo-ET by measuring different populations of structural features of interest under different conditions. Recently, quantitative analyses of membrane thickness and curvature have been the key to holistically understand biological processes as mitochondrial and bacterial division in cryo-FIB milled cellular samples. Constriction stages in mitochondrial division were identified based on membrane curvature and proximity to different cytoskeletal filaments that ultimately help fission (Mageswaran et al., 2021). Vectorization of cytoskeletal elements served to distinguish different populations of filaments based on thickness: actin, microtubules and septin filaments (Rigort et al., 2012). Further analysis of the filament populations within tomograms of wild-type *vs*. knock down Sept2 protein cells help identify the role of each filament since positioning and interactions can be then related to each division stage (Mageswaran et al., 2021). In bacteria, different division stages were established based on inner membrane closure distances measured in many dividing wild-type and cell wall biogenesis mutants. Furthermore, measurements of inner membrane–outer membrane distances were used to locate regions of cell wall thickening where a dividing cross-wall septum is formed, with important implications on the overall cell shape architecture (Navarro et al., 2021). Other examples of such cryo-ET approaches are studies concerning axon growth. Quantification of the endoplasmic reticulum in the growing shaft correlates with scarcity of ribosomes suggesting the high demand for lipid biosynthesis while low translational competence during growth (Hoffmann et al., 2021). Furthermore, the quantitative analysis of cytoskeletal elements found that the endoplasmic reticulum is tethered to microtubules through short linkers in the axons (Foster et al., 2021). In bulk STA analyses of impressively large *in situ* cryo-ET datasets (617–930 tilt-series) combined with integrative modeling of *in situ* STA structures have allowed a near-complete characterization of the nuclear pore complex and its dynamics (Mosalaganti et al., 2021; Akey et al., 2022). The combination of artificial intelligence structure-prediction methods (Baek et al., 2021; Jumper et al., 2021; Mirdita et al., 2022; Rantos et al., 2022), *in situ* cryo-ET and integrative modeling has the potential of revealing the dynamics of macromolecules when interacting with their binding partners in the cell.

Quantitative cryo-ET has also been performed in non-cellular samples. Recent studies characterize morphological differences of influenza virus by measuring size, glycoprotein spacing, and capsid organization and structure (Huang et al., 2022). Quantitative characterization of ice thickness, 3D shape, and particle distribution has also helped assessing issues (e.g., air-water interface) when performing SPA tomography (Noble et al., 2018a; Noble et al., 2018b). Furthermore, a quantitative analysis of cytoskeletal elements has provided insights into the macromolecular networks they form and their role in motility, trafficking, and cell cycle (Rigort et al., 2012; Xu et al., 2015; Anderson et al., 2017; Dimchev et al., 2021). Membrane parameterization has been also essential to achieve a quantitative ultrastructural analysis of subcellular features (Salfer et al., 2020; Barad et al., 2022). The consolidation of such analyses into integrative computational toolboxes highly facilitates the quantitative ultrastructural analysis of cryo-ET data in a time-efficient manner (Dimchev et al., 2021; Barad et al., 2022), and appears as the fittest modality for future quantitative cryo-ET research.

#### 2.1.2 Denoising

Denoising algorithms help to identify and remove noise from cryo-electron tomograms, increase SNR, and help to segment and identify features of interest (e.g., membranes, filaments) (Lucić et al., 2005). Besides its contrast improvement for visualization, such algorithms may also remove or manipulate certain amount of signal, thus, they should be used with caution and should be avoided for structure determination purposes, but are highly recommended for cellular segmentation and particle picking. Visual comparison of the original and denoised data and their respective contrast–transfer function as well as evaluation measures including the gain of SNR (e.g., peak SNR (Zhang et al., 2012) and the spectral SNR (Penczek, 2002)) as well as the inspection of the Fourier shell correlation function (van Heel and Schatz, 2005) are commonly used to ensure denoising has been properly performed (Van Heel, 1987; Frangakis, 2021).

Common filters in Fourier space are bandpass filtering and Wiener filtering by linear operation and deconvolution, respectively (Wiener, 1949; Lucić et al., 2005). Nonlinear anisotropic diffusion in real space provides signal preservation by using a variation of image gray level enhancing membranes (Frangakis and Hegerl, 2001; Fernández and Li, 2003). Bilateral denoising also uses gray scale values and their proximity to effectively suppress noise without fading high-resolution information (Jiang et al., 2003). Wavelet transformation algorithms are very efficient in high-frequency preservation since they study characteristic features inherent to signals not present in the noise (Stoschek and Hegerl, 1997). While the aforementioned filtering algorithms can be applied to 2D slices as well as the full tomogram, wavelet transformation algorithms are performed in 2D slices.

Recent approaches for image restoration and inpainting apply machine learning algorithms based on the training of deep neuronal networks. These methods need to learn what is noise and what corresponds to the sample signal from paired noisy images alone or from paired noisy and ground truth images (images with the biological sample signal of interest in the tomogram) (Buchholz et al., 2019). Topaz-Denoise is a Noise2Noise-based convolutional neural network that denoises cryo-EM images by increasing SNR, obtaining higher confidence and larger numbers when particle picking (Bepler et al., 2020). Topaz-Denoise has been shown to also work on tomograms from lamellae. IsoNet is a convolutional neuronal network system that performs image restoration and inpainting of the missing wedge in cryo-electron tomograms (Liu et al., 2021). Both algorithms are computationally expensive, needing GPUs to obtain results in a timely manner. A highly promising algorithm for image restoration of cryo-ET data is cryo-CARE, shown to significantly improve the interpretability of both cryo-EM 2D and 3D data (Buchholz et al., 2018). Such powerful denoising methods pave the way to confidently enable better segmentation of cellular features as well as a large number of particles extracted per tomogram.

Note: quality assessment of SNR-manipulated cryo-electron tomograms must be carefully inspected as these algorithms can also generate artifacts that will degrade the downstream results of segmentation and template matching since both depend on the quality of input cryo-ET data.

#### 2.1.3 Segmentation

Quantitative interpretation of tomograms requires the spatial 3D localization and geometry (shape delineation) of certain cellular substructures, e.g., organelles, membranes, filaments, and vesicles in cellular volumes. Segmentation labels such structural components present in the tomogram voxel by voxel. Such segmentations help to locate and impart an initial orientation to protein complexes of interest. Segmentation of tomograms usually requires manual annotation (tracing slice by slice) and post-processing of segmented features to analyze the curvature, shape, volume, distance, and geometry. Thus, segmentation is a very time-consuming and labor-intensive task.

Commercial software as Amira (Thermo Fisher Scientific) provides semi-automatic segmentation of biological features through thresholding algorithms, especially for cytoskeletal filaments (Rigort et al., 2012). Manual segmentation is also available in IMOD (Kremer et al., 1996). Automatically generated segmented surfaces are challenging and usually require user intervention at key steps. TomoSegMemTV focuses on automatic membrane segmentation based on tensor voting (Martinez-Sanchez et al., 2014). Additional software tools based on TomoSegMemTV automatic segmentation provide membrane geometry parameterization which is extremely useful for quantitative cryo-ET projects (Salfer et al., 2020). EMAN2 offers automated segmentation of cryo-ET data based on convolutional neural networks (Chen et al., 2017). This method can be trained to segment a variety of cellular features and tracks features slice by slice. Another potential application of segmented surfaces is the classification and initial alignment of macromolecules of interest, e.g., protein complexes inserted in membranes. One-shot learning 3D segmentation explores this possibility by performing 3D segmentation in simulated cryo-ET data based on deep learning approaches trained on a single sample set per class. Interestingly machine learning workflows aiming to combine the improvement of image contrast, 2D segmentation, refinement using reinforced learning, classification, and 3D refinement of the surface (Zhou et al., 2020) appear as promising tools for quantitative cryo-ET.

Despite the promising outputs stated previously, machine learning algorithms suffer from problems related to pixel-based density, requiring manual intervention at certain points. These algorithms are computationally intensive, needing GPU cores. The success of such algorithms is hampered by the quality of the training set, which is manually selected. High-quality subcellular segmentations are scarce, and those would be ideal to help training a neural network. For future research, it would be convenient to deposit 3D segmentations and corresponding segmented image patches in publicly available data banks.

#### 2.1.4 Template Matching

The goal of visual proteomics is to match a library of templates to cryo-electron tomograms, defining a protein atlas of the 3D positions and orientations of all macromolecules in the cell (Asano et al., 2016; Beck and Baumeister, 2016; Mahamid et al., 2016). Template matching is an image technique that performs cross-correlation of a template against an image to search for coincidences—matches. In the case of cryo-ET, the aim is to identify all occurrences of a known structure–template. The template is usually obtained from an X-ray or SPA map that is low-passed and adjusted to the resolution of the tomogram. Software tools have been developed following the standard template matching approaches. *Dynamo* and TOM packages have both MATLAB implementations to perform template matching (Frangakis et al., 2002; Nickell et al., 2005; Castaño-Díez et al., 2012). 2D high-resolution template matching has been explored to identify ribosomal populations in *Mycoplasma pneumoniae* cells and mouse fibroblasts (Rickgauer et al., 2020; Lucas et al., 2021). However, for samples thicker than 100 nm, which are the majority in cryo-ET, 2D template matching has a lower sensitivity than 3D template matching due to the background generated by neighboring and overlapping molecules.

Although computationally expensive, template matching methods are of great interest for quantitative cryo-ET since it increases both the confidence of selected features and data size. The main drawback is that the algorithms will only find what is already known—a.k.a template bias (Henderson, 2013; van Heel, 2013). To discover novel protein complexes *in situ*, new template-free algorithms appear as a promising tool in the field. These methods are based on combining pattern mining and deep learning (Xu et al., 2019, Xu et al., 2011) to identify new protein complexes, being a promising asset to generate initial templates for STA, yielding higher particle data size and the discovery of unknown macromolecular complexes and their related conformations (e.g., PySeg and DISCA) (Martinez-Sanchez et al., 2020; Zeng et al., 2021).

## 3 The Thickness Problem

Thickness influences all aspects of the cryo-ET workflow. It is a key limiting factor that has forced the field to develop methodologies such as cryo-FIB milling, cryo-electron microcopy of vitreous sections (CEMOVIS), and cryo-lift-out to visualize cells and tissues *in situ* (Figure 1). With the development of these techniques, the potential of cryo-ET has caught the attention of structural and cellular biologists (Figure 2), but also remarks its weakness as a low-throughput time-intensive pipeline and the need of extensive and expensive equipment as well as advanced trained users. Furthermore, thinning techniques denote cryo-ET as a low-throughput method, delivering low number of tomograms specifically when many mutant lines are involved. Small cryo-ET datasets compromise the quantitative characterization of a biological system.

### 3.1 The Electron Beam

Electron scattering is more frequent as the specimen becomes thicker, being the free path of a 300 kV-accelerated electron ∼ 300 nm (Grimm et al., 1996; Vulović et al., 2013). Elastic scattering (path change of emerging electrons without loss of energy) is desired for image formation since the majority of the contrast is primarily formed by interference between elastically scattered and unscattered electrons (Taylor and Glaeser, 1974; Rose, 2008; Williams and Carter, 1996). At 300 kV, the probability of inelastic scattering (path change of emerging electrons with loss and/or transfer of energy) is low, and thus, this is a desired condition when collecting tilt-series. But as the sample thickens, e.g., at higher tilt angles, the number of inelastically scattered electrons increases since the electron beam will penetrate, but will not effectively leave the sample. Those inelastic electrons are superposed on elastically scattered electrons obscuring image contrast. Energy filters help here by removing the inelastically scattered electrons increasing the quality of the image, thus, they are especially useful when imaging thick specimens as cells. Phase contrast imaging is detailed elsewhere (Zernike, 1955; Glaeser and Hall, 2011).

### 3.2 Electron Dose

The direct interaction between the electrons and the atoms of the sample damages the specimen, namely electron damage or radiation damage (Glaeser, 2008, 1971). Electron damage is more pronounced as the exposure time and beam intensity augment. This is especially important since electrons do not only alter the biological sample, e.g., breaking bonds, but they also induce motion and provoke melting of the vitrified specimen. Consequently, there is a limit on the total number of electrons tolerated by the sample: this is the electron dose. The cumulative electron dose typically ranges from 10 to 50 e^−^/Å^2^ with some cases near 70–90 e^−^/Å^2^ in SPA (Grant and Grigorieff, 2015). Importantly, dose weighting is applied to filter the radiation damage increasing SNR of micrographs (Grant and Grigorieff, 2015). In cryo-ET, the electron dose is distributed through the entire tilt-series (commonly composed of 40–60 projection images). Thus, the total electron dose (across the whole tilt-series) ranges from 90 to 240 e^−^/Å^2^ depending on the type of sample and thickness. The dose rate per projection image is ∼ 1.5—4 e^−^/Å/sec approximately, depending on the number of images composing the tilt-series. The implications of such low doses are decreasing SNR values and directed cumulative effect of electron damage that destroys high-resolution information first (Glaeser, 2016). This is especially acute in images acquired at high-tilted angles. This time-dependent loss of the signal means that the distribution of the limited electron dose across the tilt-series is a significant factor, especially when aiming for high-resolution STA. A balance must be reached between distributing more doses to the low-tilt images earlier in the acquisition sequence and ensuring that high-tilt images have sufficient signal and frequency information to contribute to the final reconstruction. Common tilt-acquisition schemes are explained in (Hagen et al., 2017). A popular scheme that preserved high-frequencies in central tilts is the Hagen or dose-symmetric scheme. This scheme is used for both high-resolution as well as cellular tomography.

### 3.3 Sample Preparation

Specimens thinner than ∼ 5–10 µm are rapidly vitrified in a cryogen (e.g., ethane) by plunge-freezing while thicker samples are vitrified under high-pressure in the presence of a cryo-protectant. Sample preparation methods for cryo-EM are detailed elsewhere (Dubochet et al., 1981, 1988; Moor, 1987). Thickness is also an important parameter to be considered when designing research strategies to answer biological questions as it affects data quality. Ice thickness and quality can be modified by optimizing blotting conditions, buffer/media composition, and grid handling. The thinner the sample, the better the image quality is achieved. Furthermore, direct cryo-ET—also known as SPA tomography and/or *in vitro* cryo-ET (only possible in samples thinner than ∼ 300 nm) —is less time consuming and more high-throughput than cryo-ET pipelines where thinning procedures are involved. Consequently, cryo-ET practitioners fervently avoid sectioning and milling methods when possible.

Currently, there are two methods that thin vitrified biological specimens: CEMOVIS and cryo-FIB milling. CEMOVIS performs cryo-sectioning by using a diamond knife under cryogenic conditions to produce ribbons composed of thin sections of the specimen collected onto EM grids (Al-Amoudi et al., 2004). Drawbacks of this technique are difficulty of handling when collecting sections as well as knife marks, crevasses, and compression artifacts induced by the cutting procedure that interfere with prospective cryo-EM/cryo-ET imaging. Cryo-focused ion beam (cryo-FIB) milling thins the samples under cryogenic conditions inside a scanning electron microscope (SEM) chamber.

### 3.4 Cryo-FIB Milling

Cryo-FIB milling uses a beam of Ga^+^ ions to ablate layers of the sample up to the desired thickness. The result is the creation of near artifact-free (curtaining and platinum deposition might affect lamellae quality when milling conditions are not optimal (Wagner et al., 2020)) thin lamellae ready to be imaged by TEM. The main steps in the cryo-FIB workflow are: 1) sample vitrification (see aforementioned section), 2) sample loading, 3) targeting of regions of interest, 4) lamellae creation by rough milling, 5) lamellae polishing, and 6) unloading (transferring lamellae to TEM microscope). For detailed cryo-FIB protocols, see (Medeiros et al., 2018; Wagner et al., 2020). The main drawbacks of cryo-FIB milling are the need of extensive additional hardware (as a cryo-FIB/SEM microscope, autogrids, liquid nitrogen dewars, and software), difficulty of targeting regions of interest, several imaging steps, and ice contamination problems whose consequences translate into low-throughput workflows. On average, a cryo-FIB milling session takes ∼ 11 h with a performance of ∼ 3–5 lamellae per hour using common best practice strategies (Table 1). Of note, cryo-FIB milling sessions are restricted to two EM grids and sessions require constant user supervision. Such drawbacks remain an obstacle for quantitative cryo-ET, although software and hardware developments aim to overcome them (Klumpe et al., 2021).

**TABLE 1:** Developments in Cryo-FIB milling.

Step	Development	Improvements	Comments	Source
Cryo-FIB milling	AutoTEM, (Aquilos, Thermo Fisher Scientific)	Unsupervised milling	Thinning may need intervention when targeting specific regions (e g., bacterial division site). Manual polishing recommended	Kuba et al. (2021)
			Lamellae thickness 50–150 nm	
			3–4 lamella per hour	
	autolamella Aquilos, Thermo Fisher Scientific	Unsupervised milling	Improvements in tracking: needs a fiducial marker (e.g., cross-shaped marker created by ion beam)	Buckley et al. (2020) www.github.com/DeMarcoLab/autolamella
			Lamellae thickness 210–250 nm	
			5 lamellae per hour	
	SmartFIB (Zeiss Microscopy GmbH, Oberkochen, Germany)	Unsupervised milling	Improvements in tracking: backlash and drift correction	Zachs et al. (2020)
			Lamellae thickness: 117–379 nm. Average = 243 nm (Manual = 258 nm)	
			16.75 min per lamella (3–4 lamella per hour)	
Cryo-lift-out	Micromanipulation	Lamellae performance on multicellular organisms and tissues	10 h per lamella	Schaffer et al. (2019); Parmenter and Nizamudeen (2021)
			Success rate ∼ 20%	
			Ongoing development	
	SerialFIB Aquilos, Thermo Fisher Scientific	Unified operational software	Includes tools for cryo-FIB milling, CLEM imaging, volume imaging automation and cryo-lift-out	Klumpe et al. (2021) https://github.com/sklumpe/SerialFIB
Cryo-transfer	Glove box transferring	Reduced frost contamination	Improve vacuum conditions for milling	Tacke et al. (2021)
	Cryo-shield and cryo-shutter	Reduced amorphous ice contamination rate	Increased session time	
			∼90% of all intact lamellae were useable for cryo-ET imaging	

### 3.5 Cryo-Lift-Out

*In situ* cryo-lift-out using in-chamber micromanipulation first extracts a small volume from a bulk sample (e.g., *C. elegans*), which is transferred onto a special EM grid for final thinning to produce a lamella via cryo-FIB milling (Mahamid et al., 2015; Parmenter and Nizamudeen, 2021; Schaffer et al., 2019). Although cryo-lift-out expands the applicability of cryo-ET to tissue and multicellular organisms (Figure 1), its full potential still depends on ongoing developments as cryo-fluorescence microscopy (cryo-FM) to target regions of interest, high-throughput freezing and milling strategies (Kelley et al., 2022) and software automation to decrease the supervised user time and increase the success rates (Klumpe et al., 2021).

### 3.6 Limitations of Sample Thinning Methods

From the aforementioned section, the thickness problem can be resolved by thinning the specimen. But two major challenges remain: targeting and sampling.

Targeting implicates imaging the region of interest. Correlative light and electron microscopy (CLEM) identifies subcellular compartments of interest for prospective EM imaging. Cryo-CLEM methods have been successfully adapted for subsequent cryo-ET of whole cells. Most completed cryo-CLEM workflows include cryo-FM, cryo-FIB and cryo-ET, involving multiple transfers between microscopes risking grid integrity and ice contamination (Kukulski et al., 2011; Arnold et al., 2016; Schorb et al., 2017). Integrated systems—a light microscope is integrated into the vacuum chamber of the SEM—, mitigates transfer risks (Faas et al., 2013; Gorelick et al., 2019), however the resolution of in-SEM chamber light microscopes is impaired by the use of long working-distance objectives. Another strategy is post-correlation on-lamella cryo-CLEM at the expense of adding extra transferring steps (Klein et al., 2021). To precisely target time events and efficiently use CLEM studies, microfluidic cryofixation during live-cell microscopy imaging appears as an interesting tool for specific specimens (Fuest et al., 2019).

Sampling refers to the cellular layers that get destroyed by cryo-FIB milling and that, by definition, cannot be imaged. An approach to be considered here is high-voltage electron microscopy (HVEM) at 1.0 MV instead of the commonly used voltage of 300 kV (Sannomiya et al., 2019). These imaging conditions are capable of providing high-contrast images with less noise of micron thick samples (Okamoto et al., 2017). Despite ongoing engineering efforts to design a compact HVEM microscope aiming to overcome the size and phase-coherence challenges, HVEM still requires an extremely large and costly TEM microscope available in very few facilities world-wide (Sannomiya et al., 2019). For future research, novel developments for HVEM could make this imaging modality a promising tool for thick samples. Another solution is cryo-scanning transmission electron tomography (cryo-STET) that allows the imaging of ∼ 3× thicker samples, albeit at a lower resolution compared to cryo-ET (Wolf and Elbaum, 2019). Again, this type of specialized EM imaging modality is not common in cryo-EM facilities due to cost–benefit reasons.

## 4 Resolution *vs*. Field of View

Ideally, cryo-ET practitioners want to image many thin specimens structurally preserved at the highest resolution possible. Structural preservation of the sample is achieved by vitrification (see Section 3.4) while the thickness problem has been covered in Section 3. What refers to ‘many’ (large N numbers, quantitative results) has been so far considered as the number of confidently extracted particles (macromolecular level) and subcellular structure such as organelles, membranes, and filaments (cellular level). Such numbers can be increased by post-acquisition computational tools for denoising (visualization), segmentation (extraction and geometry), and template matching (particle picking) as covered in Section 2. However, the number of imaged features of interest per tomogram can be increased by augmenting the field of view (FOV), in other words, by reducing magnification at the cost of resolution (Figure 2). But, as stated previously, ideally, we want to increase FOV at no resolution cost.

Camera size improvements can impact the resolution achieved but not the number of extracted particles (Kudryashev et al., 2012). To overcome such a trade-off between resolution and FOV, montage data collection schemes are being explored for cryo-ET (Peck et al., 2021; Yang et al., 2022). These approaches are especially useful for lamellae where, after a timely workflow to obtain them, the maximum information can be extracted by imaging the lamella as much as possible . These methods use beam or stage-shift and stitching of tiled acquired images at each tilt angle to obtain a composite image (Mastronarde, 2005).

Montage cryo-ET is a promising solution for imaging cryo-FIB milled lamellae as well as bridging the resolution gap and FOV trade-off (Figures 1, 2); however, it implicates increasing acquisition times per composed tilt-series and complex post-processing workflows to obtain high-quality 3D reconstructed cryo-electron tomograms. Furthermore, it still requires sacrificing part of the lamellae area for tracking and focusing. Software developments play an important role here, since integrated automated workflows will help decrease post-processing times. Likewise, camera and stage developments will help increase the speed during acquisition.

## 5 Cryo-ET Is Not Mandatory

Understanding the caveats of cryo-ET is key to keep improving the method and exploring new fields of research but also to identify biological questions that are best approached by utilizing other methods. Cryo-ET might not be the most suitable imaging technique for many biological questions. Besides its potential, cryo-ET is still ongoing major hardware and software developments to overcome inherent limitations as a time-consuming, costly, and skill-demanding method. Although such developments are extremely exciting for cryo-ET enthusiasts, and are defining cryo-ET as a new field of research on its own, those can be also frustrating for scientists driven by other research passions who see cryo-ET uniquely as a tool to answer biological questions. Therefore, it is extremely important to seriously consider the cost-benefit of applying cryo-ET to specific research projects as many limitations found in cryo-ET will greatly impact the research outcome. The main considerations for prospective cryo-ET users are: 1) sample thickness as thick samples need thinning methods prior to cryo-ET data acquisition which add another layer of complexity to the pipeline; 2) resolution needs—for high resolution, SPA methods should be considered as they are highly automated and structure determination methods are highly streamlined in the literature; 3) dynamics of the system: cryo-ET provides a static high-resolution picture of cells, organelles, and macromolecules where dynamics might be best assessed by LM studies; 4) size and *a priori* structural knowledge of the macromolecule of interest as particles can be difficult to identify in low-contrast cryo-electron tomograms (protein complexes < ∼ 300–500 kDa are challenging to study by cryo-ET alone); and 5) data size—projects prioritizing qualitative over quantitative characterization require smaller datasets unless many different conditions and/or mutants are necessary.

Many of these considerations are easily assessed by receiving inputs from cryo-EM/cryo-ET experts. Here, the increasing amount of newly inaugurated cryo-EM facilities and national centers for cryo-EM are key to exploit the applicability of cryo-EM methods. Furthermore, such cryo-EM centers provide well-documented streamlined protocols for cryo-EM sample preparation consideration as well as accessible pipelines for pre- and post-processing. Streamlined protocols for STA, segmentation algorithms, and denoising are publicly available (Navarro et al., 2018; Burt et al., 2021; Scaramuzza and Castaño-Díez, 2021; Tegunov et al., 2021) as well as common forums (https://teamtomo.org and FIB/SEM slack channel) and email lists (e.g., SerialEM, IMOD, 3DEM and *Dynamo*), which are very helpful to keep communicating challenges and discuss how to overcome them in a case-specific manner. Such streamlined protocols have also greatly benefited from the deposition of large and diverse cryo-ET datasets on databanks such as the Electron Microscopy Public Image Archive (EMPIAR). All things considered, future cryo-ET research seems extremely promising regarding both technological developments as well as biological and biomedical applications.
